# Optimal Superpixel Kernel-Based Kernel Low-Rank and Sparsity Representation for Brain Tumour Segmentation

**DOI:** 10.1155/2022/3514988

**Published:** 2022-06-24

**Authors:** Ting Ge, Tianming Zhan, Qinfeng Li, Shanxiang Mu

**Affiliations:** ^1^School of Science, Jinling Institute of Technology, Nanjing 211169, China; ^2^School of Electronic and Optical Engineering, Nanjing University of Science and Technology, Nanjing 210094, China; ^3^School of Information Engineering, Nanjing Audit University, Nanjing 211815, China

## Abstract

Given the need for quantitative measurement and 3D visualisation of brain tumours, more and more attention has been paid to the automatic segmentation of tumour regions from brain tumour magnetic resonance (MR) images. In view of the uneven grey distribution of MR images and the fuzzy boundaries of brain tumours, a representation model based on the joint constraints of kernel low-rank and sparsity (KLRR-SR) is proposed to mine the characteristics and structural prior knowledge of brain tumour image in the spectral kernel space. In addition, the optimal kernel based on superpixel uniform regions and multikernel learning (MKL) is constructed to improve the accuracy of the pairwise similarity measurement of pixels in the kernel space. By introducing the optimal kernel into KLRR-SR, the coefficient matrix can be solved, which allows brain tumour segmentation results to conform with the spatial information of the image. The experimental results demonstrate that the segmentation accuracy of the proposed method is superior to several existing methods under different indicators and that the sparsity constraint for the coefficient matrix in the kernel space, which is integrated into the kernel low-rank model, has certain effects in preserving the local structure and details of brain tumours.

## 1. Introduction

Segmenting tumour regions accurately from brain tumour MR images is conducive to the quantitative measurement and 3D visualisation of the tumours, which is of great significance for clinical treatment and medical research [1]. The goal of segmentation is to separate the lesion regions from the normal brain tissues and divide tumour regions such as edema, necrosis, and active tumours into spatially continuous regions which meet predetermined rules [2]. Since manual segmentation of 3D images by the doctor is time-consuming and unrepeatable, automatic or semiautomatic brain tumour segmentation methods are necessary.

Based on the information provided by MR images, many pixel-by-pixel classification methods have been applied to the segmentation and classification of brain images [3–10], such as multinomial logistic regression (MLR), support vector machines (SVM), and artificial neural networks (ANNs). However, classification methods that only use the grey information of the pixels obtain lower segmentation accuracy due to the influence of the noise and bias in MR images and the uncertainty of the brain tumours, while hybrid methods and kernel-based methods combining spatial and clinical information are more effective. Virupakshappa and Amarapur [11] proposed a tumour segmentation model by using FCM clustering, multiple feature extraction using Gabor Wavelets and ANN classifier, by which the proposed system accuracy increased to 85%, as evaluated on a medical MRI dataset of 40 training images and 60 test images. Kernel-based methods attempt to map linearly inseparable data to a high-dimensional feature space through a nonlinear function to make the data separable. Compared to single-kernel methods, classification methods based on multikernel strategies and MKL are more conducive to enhancing the interpretability of the decision function and expressing the attributes of the original sample space. Boughattas et al. [12] proposed a segmentation method based on multimodal MR images, in which MKL was used to associate one or more kernels with each feature and select the most relevant features to segment the edema and tumour regions. Arun and Singaravelan [13] designed a composite kernel function and applied it to the training of SVM to realise the automatic detection of brain tumours; the detection accuracy reached 93%. As classic representation learning theories, low-rank representation (LRR) and sparsity representation (SR), which mine the prior knowledge of the image by using low-rank or sparsity attributes, have been introduced into brain tumour image segmentation [14]. Due to the low-rank or sparsity constraint for the representation coefficient under the given training sample set, the structural characteristics of the image are maintained in the classification process. However, their classification performance is not satisfactory when applied to linear inseparable data as linear classifiers. For this reason, a class of classification methods based on kernel LRR (KLRR) or kernel SR (KSR) under the framework of the kernel method has emerged and achieved good results [15–20]. Such methods perform the classification by modelling the high-dimensional feature space of the image induced by the kernel function with a low-rank or sparse representation.

Superpixel segmentation clusters pixels with similar characteristics into the same region so that the local features and structural information of the image can be expressed as a whole. Compared with pixel-based image representation, superpixel-based representation is more in line with human visual cognition and involves less data redundancy. Moreover, it also provides support for extracting spatial information from an image. In order to match the spatial distribution of brain tissues and tumours, the shape of the homogeneous region should be adjusted adaptively in brain tumour segmentation. Therefore, using image features and superpixels to select homogeneous regions is a good way to overcome the drawbacks of the fixed square windows. Ge et al. [21] designed an image classifier based on multiscale superpixels and multikernel collaborative representation, in which the original grey information and the multiscale spatial features based on superpixels were combined to classify the brain tumour images.

Inspired by the above work, in this paper, we propose a segmentation approach based on the optimal superpixel kernel and KLRR-SR (OSK-KLRR-SR) for brain tumour MR images. First, the multimodal brain tumour MR images are fused, and the T1-c image is segmented by ERS [22] to generate the adaptive superpixel homogeneous regions. Second, spatial features based on the superpixels are extracted to construct the superpixel kernel, and the optimal superpixel kernel is selected by representative MKL [23]. Finally, the brain tumour image is modelled by KLRR-SR, in which the coefficient matrix is solved by introducing the optimal superpixel kernel so as to extract the regions of necrosis, enhanced tumours, and edema, respectively. The advantages of the proposed method are as follows: (1) In addition to taking advantage of kernel methods, the proposed KLRR-SR model preserves both the overall structure and local details in the high-dimensional feature space of the image through the joint constraints of LRR and SR, thereby improving the representation accuracy of the image. (2) By considering spatial features based on the superpixel homogeneous region, the superpixel kernel adaptively learns the high-dimensional manifold features of each class of samples in brain tumour images, which measures the pairwise similarity of samples more accurately. (3) The MKL method resolves the difficulties of multiscale feature learning and adaptive parameter determination in traditional kernel methods. Experiments on MICCAI BraTS 2013 dataset show that the segmentation results of the proposed method are close to the standard results, and the isolated region, the slender topology, and the boundaries of tumour are well preserved due to the sparsity constraints incorporated into the KLRR model. The quantitative comparison of the segmentation accuracy for the regions of necrosis and enhanced tumours under different indicators shows that the proposed method has certain advantages in brain tumour segmentation compared with several existing methods.

## 2. Materials and Methods

### 2.1. Superpixel Segmentation

To accurately extract the spatial structure features of brain tumours and brain tissues, superpixel segmentation is adopted to adaptively obtain the uniform regions in the image. Here, we use the entropy rate superpixel method (ERS) [22], which is based on graph partitioning and entropy rate, to perform image segmentation.

We first map the input image *X*={*x*_1_,  *x*_2_,…,  *x*_*N*_} to an undirected graph *G*=(*V*, *E*), where *V* is the vertex set consisting of all the pixels in the image and *E* is the edge set. Further, *e*_*ij*_ ∈ *E* represents the edge connecting adjacent pixels *x*_*i*_ and *x*_*j*_, and *ω*_*ij*_ is the corresponding weight, which is used to reflect the similarity between *x*_*i*_ and *x*_*j*_. The superpixel segmentation for *X* can then be obtained by dividing the graph *G*, that is, selecting a subset *A* ⊂ *E* to form an undirected graph *G*′=(*V*, *A*), which contains *P* subgraphs. The objective function of ERS is

(1) $$\begin{array}{c} \underset{A}{\max}H\left(A\right)+\lambda B\left(A\right)\\ \text{s.t. }A\;\subseteq \;E,\;N_{A}\geq P, \end{array}$$

where *A* is the selected edge set and *λ* is the weight factor. *H*(*A*) is the entropy rate of a random walk on the graph, which is used to prefer the formation of compact and homogeneous clusters, and *B*(*A*) is the balancing term, which is used to induce clusters with similar sizes. It is proven that both the entropy rate and the balancing term are monotonically increasing submodular functions under the proposed graph construction; therefore, the objective function is also submodular and monotonically increasing. Furthermore, by introducing a matroid for optimisation, the solution of equation (1) presents an effective greedy algorithm.

The ERS algorithm is stable, and the generated superpixels are not only controllable in number but also have a good boundary fit, which helps to maintain the target structures in the image.

### 2.2. Optimal Superpixel Kernel Based on MKL

Let *X*={*x*_1_,  *x*_2_,…,  *x*_*N*_} ∈ *R*^*L*×*N*^ represent the fusion data of multimodal brain tumour MR images, in which *x*_*i*_ ∈ *R*^*L*^ represents the *i-*th pixel feature, and *N* is the total number of pixels. Use ERS to perform superpixel segmentation on the T1-c image, and let {*X*_1_, *X*_2_,…, *X*_*P*_} be the segmentation results of *X*, in which *X*_*i*_ is the *i-*th superpixel, and *P* is the number of superpixels. Suppose there exists a nonlinear function *ϕ* that maps the pixel feature (i.e., a testing sample or a training sample) to the high-dimensional Hilbert space. Set *x*_*k*_^(*i*)^ as the *k*-th pixel in *X*_*i*_. Its spatial feature is given by the superpixel-based mean filtering form as

(2) $$\begin{array}{c} \phi^{\prime }\left(x_{k}^{\left(i\right)}\right)=\frac{1}{N_{i}}\sum_{m=1}^{N_{i}}\phi \left(x_{m}^{\left(i\right)}\right),\;x_{m}^{\left(i\right)}\in X_{i},\;m=1,2,\ldots N_{i}, \end{array}$$

where *x*_*m*_^(*i*)^ is the *m*-th pixel in *X*_*i*_, *N*_*i*_ is the number of pixels located in *X*_*i*_, and ∑_*i*=1_^*P*^*N*_*i*_=*N*. The superpixel kernel between *x*_*k*_^(*i*)^ and *x*_*s*_^(*j*)^ can be written as

(3) $$\begin{array}{c} K_{SP}\left(x_{k}^{\left(i\right)},x_{s}^{\left(j\right)}\right)=\langle \phi^{\prime }\left(x_{k}^{\left(i\right)}\right),\phi^{\prime }\left(x_{s}^{\left(j\right)}\right)\rangle \\ =\langle \frac{1}{N_{i}}\sum_{m=1}^{N_{i}}\phi \left(x_{m}^{\left(i\right)}\right),\frac{1}{N_{j}}\sum_{n=1}^{N_{j}}\phi \left(x_{n}^{\left(j\right)}\right)\rangle \\ =\frac{1}{N_{i}N_{j}}\sum_{m=1}^{N_{i}}\sum_{n=1}^{N_{j}}\langle \phi \left(x_{m}^{\left(i\right)}\right),\phi \left(x_{n}^{\left(j\right)}\right)\rangle \\ =\frac{1}{N_{i}N_{j}}\sum_{m=1}^{N_{i}}\sum_{n=1}^{N_{j}}\kappa \left(x_{m}^{\left(i\right)},x_{n}^{\left(j\right)}\right), \end{array}$$

where *κ*(*x*_*m*_^(*i*)^, *x*_*n*_^(*j*)^) is the basic kernel function and is taken to be the Gaussian RBF kernel, which is given as

(4) $$\begin{array}{c} \kappa \left(x_{m}^{\left(i\right)},x_{n}^{\left(j\right)}\right)=\exp\left(-\frac{{\left\|x_{m}^{\left(i\right)}-x_{n}^{\left(j\right)}\right\|}_{2}^{2}}{2\sigma^{2}}\right),\;\sigma >0. \end{array}$$

Considering the complex structures and fuzzy boundaries in the brain tumour MR images, multiscale kernels are used to measure the similarity between samples from different categories. Select *M* kernel scales *σ*_min_=*σ*_1_ < *σ*_2_ < ⋯<*σ*_*M*_=*σ*_max_ within the range [*σ*_min_, *σ*_max_]. Based on equation (3), the Gram matrixes *G*_*i*_ under the scale *σ*_*i*_ is as follows:

(5) $$\begin{array}{c} G_{i}=\left[\begin{array}{cccc} K_{SP}\left(x_{1},x_{1}\right) & K_{SP}\left(x_{1},x_{2}\right) & \cdots & K_{SP}\left(x_{1},x_{N}\right)\\ K_{SP}\left(x_{2},x_{1}\right) & K_{SP}\left(x_{2},x_{2}\right) & \cdots & K_{SP}\left(x_{2},x_{N}\right)\\ \vdots & \vdots & \vdots & \vdots \\ K_{SP}\left(x_{N},x_{1}\right) & K_{SP}\left(x_{N},x_{2}\right) & \cdots & K_{SP}\left(x_{N},x_{N}\right) \end{array}\right],\;i=1,2,\ldots ,M. \end{array}$$

Let *v*(*G*_*i*_) ∈ *R*^*N*^2^^ denote the column vector generated by vectorizing the matrix *G*_*i*_ in a fixed order, and we can obtain a new expression in the form of *M* kernel matrixes *G*_*SP*_=[*v*(*G*_1_), *v*(*G*_2_),…,*v*(*G*_*M*_)]^*T*^=[g_1_, g_2_,…, g_*N*^2^_] ∈ *R*^*M*×*N*^2^^, in which *g*_*i*_ is an *M*-dimensional column vector, *i*=1,2,…, *N*^2^. In order to find the low-dimensional linear subspace in the kernel matrix group, we construct the following loss function [23]:

(6) $$\begin{array}{c} L\left(W,Z\right)={\left\|G_{SP}-WZ\right\|}_{F}^{2}\\ =\sum_{i=1}^{N^{2}}\sum_{j=1}^{p}{\left(g_{ij}-\sum_{t=1}^{p}w_{it}z_{tj}\right)}^{2}, \end{array}$$

where *W*=(*w*_1_, *w*_2_,…, *w*_*p*_) ∈ *R*^*M*×*p*^ is the projection matrix whose columns *w*_1_, *w*_2_,…, *w*_*p*_ are the bases of a *p*-dimensional linear subspace, *Z* ∈ *R*^*p*×*N*^2^^ is the projected matrix onto the linear subspace spanned by *W*, and *g*_*ij*_, *w*_*ij*_, and *z*_*ij*_ are the elements of *G*_*SP*_, *W*, and *Z*, respectively. According to the projection theorem, equation (6) will be minimized by setting *Z*=*W*^*T*^*G*_*SP*_, and its dual problem is as follows:

(7) $$\begin{array}{c} \left\{\begin{array}{c} \underset{W}{\arg\;\max}{\left\|W^{T}\sum_{G_{SP}}W\right\|}_{F}=\underset{W}{\arg\;\max}{\left\|W^{T}G_{SP}\right\|}_{F},\\ \text{s.t. }W^{T}W=I_{p}, \end{array}\right. \end{array}$$

where ∑*G*_*SP*_=*G*_*SP*_*G*_*SP*_^*T*^ and *I*_*p*_ is the *p* × *p* identity matrix. By setting *p*=1 and solving equation (7) by singular value decomposition, we can obtain the projection vector *W*^*∗*^=[*w*_11_^*∗*^, *w*_21_^*∗*^,…,*w*_*M*1_^*∗*^]^*T*^ with maximum variance direction, which is just the optimal weight vector of the kernel function. As a result, the optimal kernel function is given by

(8) $$\begin{array}{c} K^{*}=\sum_{m=1}^{M}w_{m1}^{*}\kappa_{m}. \end{array}$$

Referring to equation (3), the optimal superpixel kernel can be written as

(9) $$\begin{array}{c} K_{SP}^{*}\left(x_{k}^{\left(i\right)},x_{s}^{\left(j\right)}\right)=\frac{1}{N_{i}N_{j}}\sum_{m=1}^{N_{i}}\sum_{n=1}^{N_{j}}K^{*}\left(x_{m}^{\left(i\right)},x_{n}^{\left(j\right)}\right). \end{array}$$

The steps to generate the optimal superpixel kernel based on MKL are given by Algorithm 1.

### 2.3. OSK-KLRR-SR Classifier

The greyscale distribution of the MR image is not uniform due to factors in the imaging process such as the offset field. When the variation range of the pixel grayscale is close to the image contrast, the accuracy of classification methods based on the statistical characteristics of the greyscale will be reduced. For this reason, it is necessary to mine the image features in the spectral kernel space and to build a more robust classification model by using the structure prior of the image. Classification methods based on KLRR or KSR combine the linear separability of the high-dimensional feature space induced by the kernel function with the advantage of LRR or SR in preserving the structural features of the data under the framework of the kernel method. Note that the high-dimensional features used in KLRR and KSR for image classification are only based on disordered pixels without considering the spatial information. In this paper, we propose a brain tumour image classification model based on the joint representation of KLRR and KSR, in which the optimal superpixel kernel is introduced to solve the coefficient matrix so that the classification process can be integrated with the image spatial features. The optimal superpixel kernel generation from the superpixel homogeneous region and MKL improves the similarity measurement accuracy of samples. The joint constraints of KLRR-SR preserve the local features in the image as well as the overall structure, which is helpful to improve the image representation accuracy. Therefore, the performance of the proposed method in promoting the segmentation accuracy of brain tumour regions can be expected.

Let *D*=[*d*_1_, *d*_2_,…, *d*_*T*_] be the dictionary constructed by the training samples, in which d_*i*_ with *i*=1,2,…*T* represents the *i*-th training sample. By defining the mapping function Φ(*X*)={*ϕ*(*x*_1_), *ϕ*(*x*_2_),…, *ϕ*(*x*_*N*_)} and Φ(*D*)={*ϕ*(*d*_1_), *ϕ*(*d*_2_),…, *ϕ*(*d*_*T*_)}, the classification model based on the joint representation of KLRR-SR can be constructed as follows:

(10) $$\begin{array}{c} \underset{A}{\min}\frac{1}{2}{\left\|\Phi \left(X\right)-A\Phi \left(D\right)\right\|}_{F}^{2}+\lambda {\left\|A\right\|}_{*}+\alpha {\left\|A\right\|}_{1}, \end{array}$$

where *A* is the coefficient matrix and *λ* and *α* are the regulatory factors that adjust the weights of the low-rank and sparse term, respectively. The larger their values, the stronger the low-rank and sparse constraints on *A*.

After solving for the optimal solutions *A*^*∗*^ corresponding to *A* in equation (10), the classification function is

(11) $$\begin{array}{c} \text{class}\left(x_{i}\right)=\underset{c=1,2,\ldots C}{\arg\;\min}{\left\|\phi \left(x_{i}\right)-\Phi \left(D\right)\delta_{c}\left(a_{i}^{*}\right)\right\|}_{F}^{2}, \end{array}$$

where *c*={1,2,…, *C*} is the class label set and *a*_*i*_^*∗*^ is the *i*-th column vector of *A*^*∗*^. *δ*_*c*_(*a*_*i*_^*∗*^) represents an indicator operation that zeroes out all elements of *a*_*i*_^*∗*^ that do not belong to the class *c*.

By introducing the optimal superpixel kernel in equation (9), equation (10) can be transformed into the following inner-product form

(12) $$\begin{array}{c} \underset{A}{\min}\frac{1}{2}tr\left(A^{T}VA\right)-tr\left(A^{T}U\right)+\lambda {\left\|A\right\|}_{*}+\alpha {\left\|A\right\|}_{1}+C, \end{array}$$

where *C* represents a constant term. *V* and *U* are the matrixes with elements *V*_*ij*_=*K*_*SP*_^*∗*^(*d*_*i*_, *d*_*j*_) and *U*_*ij*_=*K*_*SP*_^*∗*^(*d*_*i*_, *x*_*j*_), respectively. Thus, the classification function can be rewritten as follows:

(13) $$\begin{array}{c} \text{class}\left(x_{i}\right)=\underset{c=1,2,\ldots C}{\arg\;\min}\delta_{C}^{T}\left(a_{i}^{*}\right)V\delta_{c}\left(a_{i}^{*}\right)-2\delta_{C}^{T}\left(a_{i}^{*}\right)U. \end{array}$$

Equation (12) is a convex problem, which can be solved by the alternating direction method of multipliers (ADMM) [24]. To make the objective function separable, we introduce auxiliary variables *E* and *F*, such that equation (12) can be rewritten as

(14) $$\begin{array}{c} \left\{\begin{array}{c} \underset{A}{\min}\frac{1}{2}tr\left(A^{T}VA\right)-tr\left(A^{T}U\right)+\lambda {\left\|E\right\|}_{*}+\alpha {\left\|F\right\|}_{1},\\ \text{s.t. }E=A,\\ F=A. \end{array}\right. \end{array}$$

The augmented Lagrange function is

(15) $$\begin{array}{c} L_{\mu }\left(A,E,F,\mu \right)=\frac{1}{2}tr\left(A^{T}VA\right)-tr\left(A^{T}U\right)+\lambda {\left\|E\right\|}_{*}+\alpha {\left\|F\right\|}_{1}\\ +tr\left(Y_{1}^{T}\left(A-E\right)\right)+tr\left(Y_{2}^{T}\left(A-F\right)\right)+\frac{\mu }{2}\left({\left\|A-E\right\|}_{F}^{2}+{\left\|A-F\right\|}_{F}^{2}\right), \end{array}$$

where *Y*_1_ and *Y*_2_ are Lagrange multipliers and *μ* is a penalty factor. When solving the above-unconstrained optimisation problem, ADMM uses a strategy of alternately updating one variable while fixing the remaining variables. The variable updating strategy is given as

(16) $$\begin{array}{c} E^{*}=\underset{E}{\arg\;\min}\lambda {\left\|E\right\|}_{*}+tr\left(Y_{1}^{T}\left(A-E\right)\right)+\frac{\mu }{2}{\left\|A-E\right\|}_{F}^{2}\\ =\underset{E}{\arg\;\min}\frac{\lambda }{\mu }{\left\|E\right\|}_{*}+\frac{1}{2}{\left\|E-A-\left(\frac{Y_{1}}{\mu }\right)\right\|}_{F}^{2}, \end{array}$$

(17) $$\begin{array}{c} F^{*}=\underset{F}{\arg\;\min}\alpha {\left\|F\right\|}_{1}+tr\left(Y_{2}^{T}\left(A-F\right)\right)+\frac{\mu }{2}{\left\|A-F\right\|}_{F}^{2}\\ =\underset{F}{\arg\;\min}\frac{\alpha }{\mu }{\left\|F\right\|}_{1}+\frac{1}{2}{\left\|F-A-\left(\frac{Y_{2}}{\mu }\right)\right\|}_{F}^{2},\\ +\frac{\mu }{2}\left({\left\|A-E\right\|}_{F}^{2}+{\left\|A-F\right\|}_{F}^{2}\right). \end{array}$$

(18) $$\begin{array}{c} A^{*}=\underset{A}{\arg\;\min}\frac{1}{2}tr\left(A^{T}VA\right)-tr\left(A^{T}U\right)+tr\left(Y_{1}^{T}\left(A-E\right)\right)+tr\left(Y_{2}^{T}\left(A-F\right)\right)\\ +\frac{\mu }{2}\left({\left\|A-E\right\|}_{F}^{2}+{\left\|A-F\right\|}_{F}^{2}\right). \end{array}$$

The optimal solutions of equations (15) through (17) are as follows:

(19) $$\begin{array}{c} E^{*}=T_{\lambda /\mu }\left(\frac{A+Y_{1}}{\mu }\right)=P\Theta_{\lambda /\mu }\left(\Sigma \right)Q^{T},\\ F^{*}=\Theta_{\alpha /\mu }\left(\frac{A+Y_{2}}{\mu }\right),\\ A^{*}={\left(V+2\mu I\right)}^{-1}\left(U+\mu \left(E+F-\left(\frac{Y_{1}+Y_{2}}{\mu }\right)\right)\right), \end{array}$$

where *P*(Σ)*Q*^*T*^ is the singular value decomposition result of *A*+*Y*_1_/*μ* and Θ is the soft thresholding operator.

In summary, the general algorithm for the OSK-KLRR-SR classifier for brain tumour segmentation is given as Algorithm 2.

## 3. Results and Discussion

**Figure 1:**
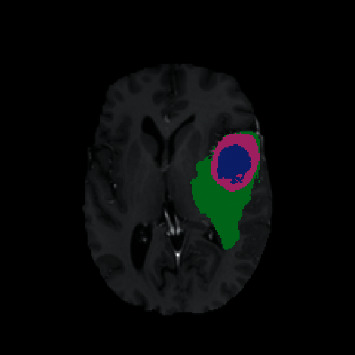
Standard manual segmentation result.

To evaluate the effectiveness of the proposed method, we performed experiments on the BraTS 2013 dataset [25, 26] to extract the three brain tumour regions of necrosis, enhanced brain tumours, and edema, respectively. The proposed segmentation model was built upon the training dataset provided by BraTS 2013, which consists of the MR images from 30 brain glioma cases (20 high-grade glioma cases and 10 low-grade glioma cases) with standard segmentation results available. The standard segmentation results were annotated by a trained team of radiologists, altogether comprising seven radiographers in Bern, Debrecen, and Boston and containing four marked tumour regions of necrosis, enhancing core, nonenhancing solid core, and edema. As shown in Figure 1, the blue area is necrosis, the pink area is enhanced tumours, and the green area is edema. All the images in the dataset include four modalities of T1, T2, T1-c, and Flair and have been registered and shelled in advance.

### 3.1. Parameter Analysis

#### 3.1.1. The Number of Superpixels

**Figure 2:**
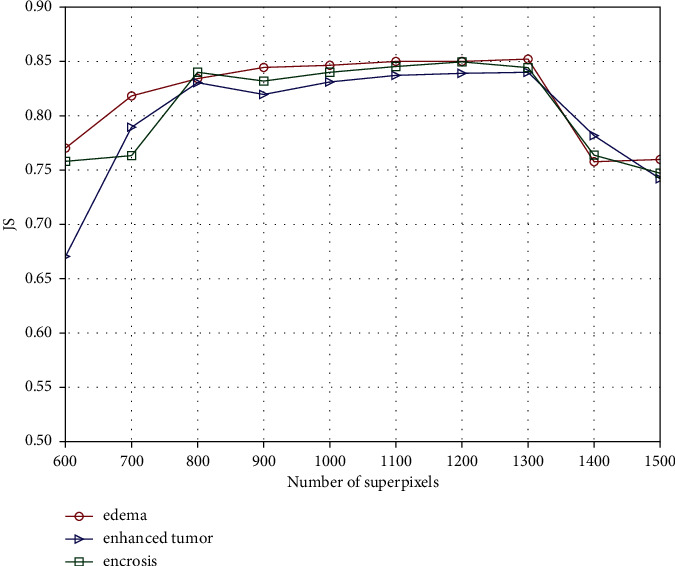
Correlation between the segmentation accuracy and the number of superpixels.

In order to study the influence of the number of superpixels on brain tumour segmentation accuracy, the Jaccard Similarity (JS) of the brain tumour regions obtained by the proposed method with different numbers of superpixels is given in Figure 2. The segmentation performance is not satisfactory when the number is too large or too small. The reason is that the regional uniformity will become larger when the number of superpixels is too large, which may cause the pixels contained in a single superpixel to come from different categories. On the other hand, the performance of the spatial constraints will be reduced when the number of superpixels is too small, resulting in a decrease in the classification accuracy. The proposed method achieves better segmentation performance when the number of superpixels is in the range [800, 1200].

#### 3.1.2. Parameter *λ*

**Figure 3:**
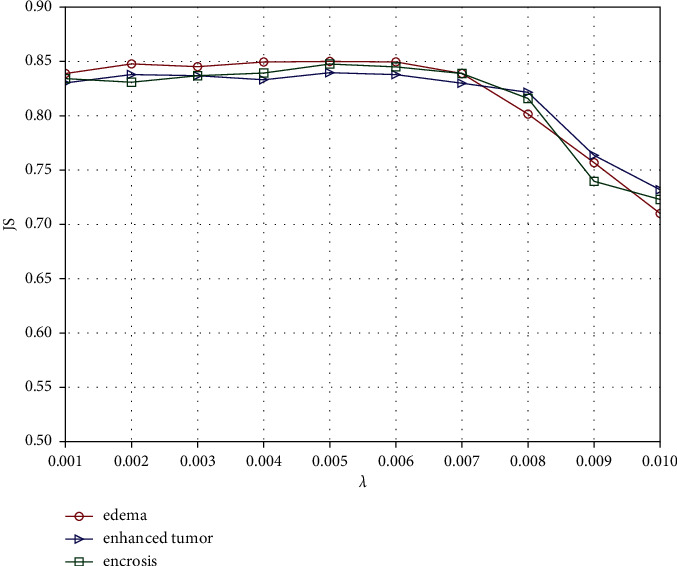
Correlation between the segmentation accuracy and the parameter *λ*.

The parameter *λ* is the weight to adjust the low-rank term in equation (10).  Figure 3 shows the segmentation accuracy with different values of *λ*. From the results, we see that JS performs better when the value is in the range of [0.001, 0.007] and decreases rapidly when the value exceeds 0.007. This is because the larger the value of *λ*, the stronger the constraint effect of the low-rank term. An overly strong low-rank constraint forces pixels belonging to different categories to be classified into the same class, which will reduce the classification performance of the first item in equation (10). In our experiments, the value of *λ* is set to 0.005.

#### 3.1.3. Parameter *α*

**Figure 4:**
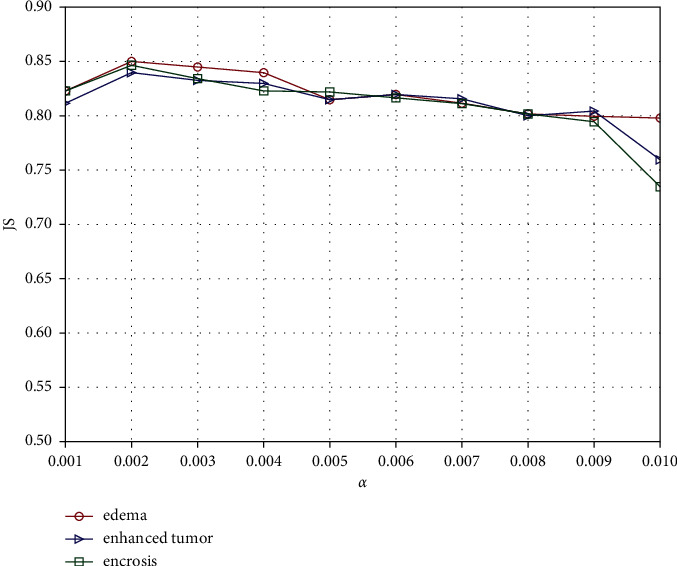
Correlation between the segmentation accuracy and the parameter *α*.

The parameter *α* refers to the weight of the sparse term in equation (10). The correlation diagram between its different values and the segmentation accuracy is drawn in Figure 4. We see that JS is satisfactory when the value of *α* is in the range of [0.001, 0.009] and drops rapidly when its value exceeds 0.009. This is because a larger value of *α* will enhance the sparsity constraint, and an excessively strong sparsity constraint will lead to more isolated points in the segmentation results. In our experiments, the value of *α* is set to 0.002.

#### 3.1.4. The Number of Training Samples

**Figure 5:**
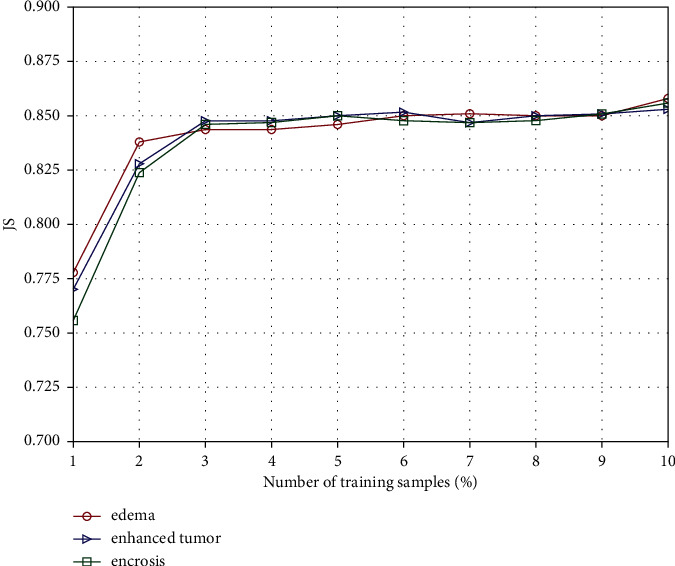
Correlation between the segmentation accuracy and the number of training samples.

The image dictionary required in the proposed method is constructed by randomly selecting marked pixels from each category, with the remaining unselected pixels used as testing samples to evaluate the segmentation performance of the method. Different numbers of training samples are adopted to study its influence on the segmentation accuracy of the proposed method in Figure 5. From the results, the segmentation accuracy on the testing samples is high and is relatively stable when 3% of the total pixels in the image are selected as training samples, which demonstrates that the proposed method can achieve better classification results under the small training set.

### 3.2. Segmentation Results on Brain Tumour Regions

**Figure 6:**
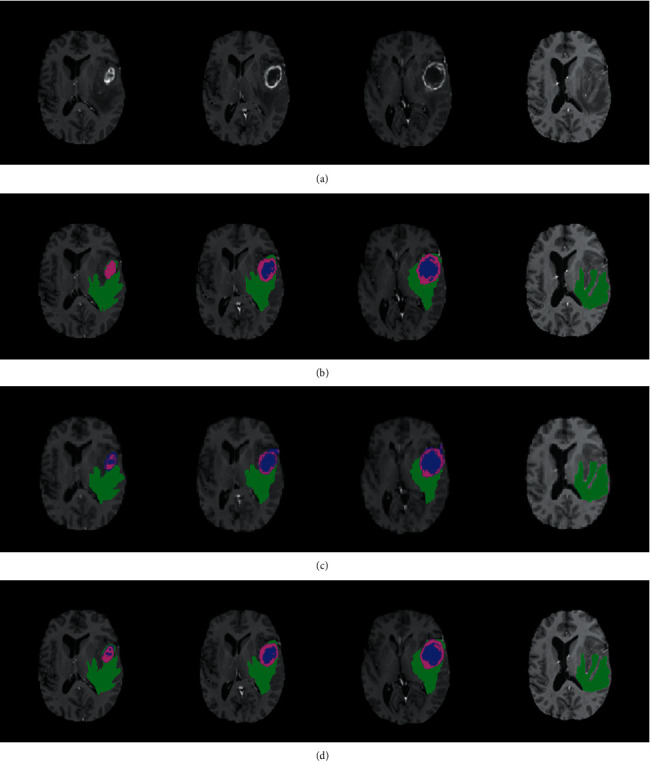
Segmentation results of a high-grade glioma case. (a) Original brain tumour images of different slices in T1-c modality. (b) Standard manual segmentation results. (c) Segmentation results obtained by OSK-KLRR. (d) Segmentation results obtained by OSK-KLRR-SR.

**Figure 7:**
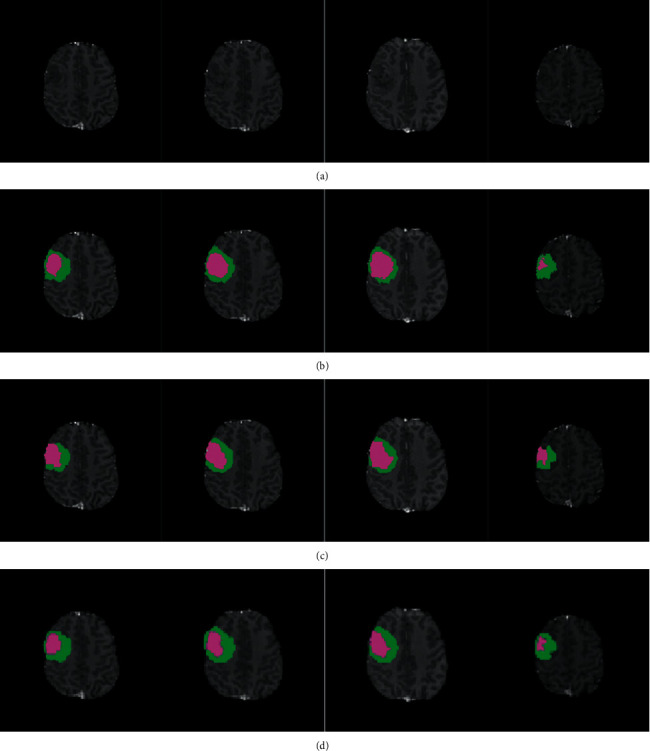
Segmentation results of a low-grade glioma case. (a) Original brain tumour images of different slices in T1-c modality. (b) Standard manual segmentation results. (c) Segmentation results obtained by OSK-KLRR. (d) Segmentation results obtained by OSK-KLRR-SR.

Two groups of segmentation results, on a high-grade case and a low-grade glioma case, are shown in Figures 6 and 7, respectively. Among these images, the first line gives the original brain tumour images of different slices in T1-c modality, the second line gives the standard manual segmentation results, the third line gives the segmentation results obtained by the optimal superpixel kernel-based KLRR (OSK-KLRR) classifier, and the fourth line gives the segmentation results obtained by OSK-KLRR-SR. It can be seen from the figures that both segmentation results obtained by OSK-KLRR and OSK-KLRR-SR are close to the standard manual segmentation results. In addition, OSK-KLRR-SR is superior to OSK-KLRR in maintaining the local structures and details of the image, such as the isolated area and slender topology inside the brain tumour and the tumour boundaries due to the sparsity constraints for the coefficient matrix. As such, the segmentation results obtained by OSK-KLRR-SR are closer to the standard segmentation results.

For quantitative analysis, Table 1 lists the segmentation accuracy for the lesion regions of necrosis and enhanced tumours under different indicators obtained by the proposed method and other methods. From the results, we see that the segmentation accuracy obtained by OSK-KLRR-SR ranks first in the two types of lesion regions, indicating that the proposed method has certain advantages in the field of brain tumour segmentation. Note that OSK-KLRR-SR achieves higher performance than OSK-KLRR, which verifies that the sparsity constraint for the coefficient matrix in the kernel space helps preserve the local structure and details of brain tumours.

## 4. Conclusion

In this paper, a segmentation method based on the optimal superpixel kernel and KLRR-SR for brain tumour MR images is proposed. First, the T1-c image is segmented by ERS to generate uniform regions, and the superpixel kernel is constructed based on image spatial features. Then, MKL is used to learn the optimal weight vector for generating the optimal superpixel kernel. Finally, KLRR-SR is adopted to model the brain tumour image, and the representation coefficient matrix is solved by introducing the optimal superpixel kernel so as to realise the extraction of regions of necrosis, enhanced tumours, and edema, respectively. By combining the linear separability of the high-dimensional space with the advantages of LRR and SR in preserving the structural features of the image, the representation accuracy of the brain tumour image is improved. In addition, the optimal superpixel kernel based on the image spatial information and MKL adaptively learns the high-dimensional manifold features of each class of samples in brain tumour image, thus improving the accuracy of feature extraction. Quantitative comparison of segmentation accuracy under different indicators indicates that the proposed OSK-KLRR-SR classifier provides improved performance over several existing methods and shows certain advantages in preserving the boundary and detail features of brain tumours as well as the overall structures of the image.

## Figures and Tables

**Algorithm 1:**
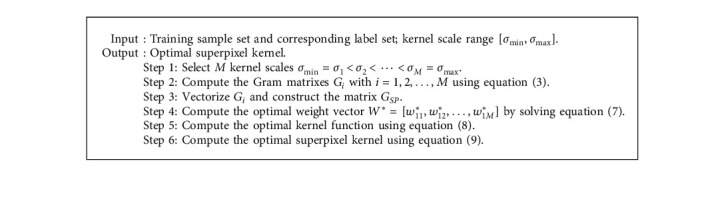
Optimal superpixel kernel generation based on MKL.

**Algorithm 2:**
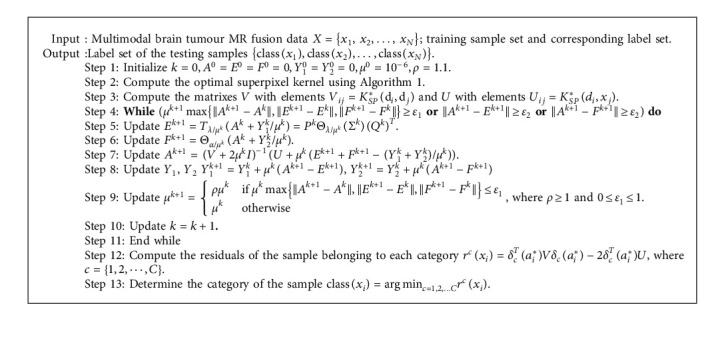
The algorithm of the OSK-KLRR-SR classifier.

**Table 1:** Comparison of segmentation accuracy of different methods under different indicators.

Methods	Dice		PPV		Sensitivity	
	Necrotic	Enhanced	Necrotic	Enhanced	Necrotic	Enhanced
Zhao et al. [27]	0.84	0.77	0.87	0.76	0.82	0.80
Havaei et al. [28]	0.79	0.73	0.79	0.68	0.79	0.80
Pereira et al. [29]	0.83	0.77	0.87	0.74	0.83	0.81
Tustison et al. [30]	0.78	0.74	0.74	0.69	**0.88**	0.83
Kwon et al. [31]	0.83	0.72	**0.90**	0.74	0.78	0.72
OSK-KLRR	0.85	0.78	0.89	0.80	0.87	**0.85**
OSK-KLRR-SR	**0.86**	**0.79**	**0.90**	**0.81**	**0.88**	**0.85**

## Data Availability

The data used to support the findings of this paper are from open datasets; please visit https://www.smir.ch/BRATS/Start2013.
